# Adopting a Point-of-Care Model for Genetic Testing in Children With Developmental Delays: A Case Report

**DOI:** 10.7759/cureus.64589

**Published:** 2024-07-15

**Authors:** Katherine A Kessler, Mandeep Kaur, Elizabeth Shaffer

**Affiliations:** 1 Psychiatry, Cape Fear Valley Health, Fayetteville, USA; 2 Child and Adolescent Psychiatry, Cape Fear Valley Health, Fayetteville, USA

**Keywords:** point of care testing, genetic testing, child and adolescent psychiatry, autism spectrum disorder (asd), autism screening

## Abstract

It is the current consensus amongst the psychiatric community that children undergoing evaluation for developmental delays and/or autism spectrum disorder (ASD) should be offered genetic testing early in the diagnostic process. Identifying genetic abnormalities can provide insight into patient prognosis and may reveal other medical complications that could arise throughout a patient’s life. Despite these recognized benefits, genetic testing is often delayed or not offered and therefore deprives families of valuable knowledge about their child’s future health outcomes. We present a case of a six-year-old patient who presented to our child and adolescent psychiatry office for behavioral concerns. She had received an ASD diagnosis years prior to presentation, but for unknown reasons, genetic testing had never been pursued. Genetic testing was obtained in our office, and the results revealed three different mutations that were linked to ASD and various other medical complications including epilepsy. With this knowledge, the patient’s family gained important insight into their child’s prognosis. This case highlights the necessity for adopting a point-of-care testing (POCT) model when evaluating children with developmental delays and/or ASD. Through this model, genetic testing would be offered to families during the initial visit for these patients. This would help streamline this process and allow for more widespread detection of genetic disorders linked to ASD and coexisting medical sequelae. Having this knowledge would empower families with a better understanding of their child’s condition and would allow families to work together with providers to determine the best possible treatment plan.

## Introduction

The rate of pediatric patients receiving a diagnosis for a developmental disability is steadily rising in the United States (US). Recent studies have estimated that roughly one in six children in the age range of 3-17 years currently meet diagnostic criteria for a developmental disability, examples of which include attention-deficit/hyperactivity disorder (ADHD), autism spectrum disorder (ASD), and intellectual disability (ID) [1]. Similarly, the overall prevalence of ASD in this population has steadily increased over recent decades, and it is estimated that roughly one in 36 children in the US meet the diagnostic criteria for ASD [1,2]. ASD is characterized by hallmark features which include impairments in social communication, difficulty interpreting non-verbal cues, repetitive, restricted, and fixated behaviors as well as abnormal reactions to sensory cues [3]. Typically, symptoms of ASD become recognizable during the second year of a child’s life when caregivers notice deficits in language development along with poor socialization skills [3]. Although symptomatic severity in children with ASD is highly variable, the diagnosis is often made on a clinical basis by a child’s primary care provider. As the incidence of ASD has dramatically increased over the last couple of decades, numerous studies have been conducted looking into both potential genetic and environmental contributors to ASD. Both twin studies in addition to population-based cohort studies have proven that there is a strong genetic influence on the development of ASD [4]. 

It is the current consensus amongst the medical community that children meeting diagnostic criteria for ASD should undergo formal genetic evaluation for numerous reasons [5,6]. Firstly, studies have proven that ASD is heritable, and it is estimated that the recurrence risk of ASD amongst siblings may be as high as 19% [7]. Discovering a potentially inherited genetic disorder in one sibling can prompt concurrent testing for other family members and may impact future family planning. Another benefit is that receiving a formal genetic-based diagnosis for ASD can make obtaining coverage for social resources or services easier by providing evidence of the underlying biological basis of the condition. However, availability and coverage for services may vary depending on the location and insurance policies. Finally, there are a multitude of genetic disorders that can cause both ASD and sometimes other serious medical comorbidities. Studies have shown over 95% of individuals with an ASD diagnosis have at least one co-occurring medical or behavioral condition [8]. For instance, epilepsy is frequently seen to coexist with ASD, and some more notable neurodevelopmental disorders where this is the case include Rett syndrome, Fragile X syndrome, and tuberous sclerosis complex [9]. Methods such as chromosomal microarray and whole exome sequencing (WES) are frequently utilized for uncovering genetic mutations that lead to ASD and associated medical conditions [10]. If genetic testing is performed early in the ASD workup, it may reveal information about potential medical conditions that patients have yet to experience. Having this information empowers patients and their families to seek specialized care and helps personalize the entire treatment process.

In the past, receiving a formal genetic workup was a limiting factor as this required a referral to a clinical geneticist for formal workup and subsequent genetic counseling [5]. This was especially challenging for families facing financial difficulties or for those residing in more rural areas with limited resources. In recent years, the production of in-office kits has simplified accessing genetic testing. Kits allow for buccal swab collection that can be performed in the office and sent immediately for referral, allowing patients and providers to obtain results within a few weeks [11]. With this increased ease of access and the numerous benefits that arise from early genetic testing, early genetic testing in these patients should be emphasized and made a gold standard by all pediatricians and primary care physicians. We propose that the psychiatric and primary care community adopt a point-of-care testing (POCT) approach to genetic evaluation in patients with high clinical suspicion for ASD. This would help streamline the process and provide families with early access to information about their child’s future medical outcomes. We hereby present a case that emphasizes the importance of early genetic testing in pediatric patients with neurodevelopmental disorders.

## Case presentation

A six-year-old female was referred to our outpatient child and adolescent psychiatry clinic for evaluation. The patient’s pediatrician recommended a formal psychiatric workup after parents complained of a recent escalation in behavioral outbursts, aggression, and overall irritability. The patient had been diagnosed with ASD at age three by her pediatrician based on clinical presentation. She had never undergone neuropsychiatric testing to help confirm the diagnosis. Her past medical history was significant for speech delay, visual deficits, and poor dentition (evidenced by entire right upper molars being fully capped). Other notable physical findings included hypertelorism and small stature. Past workup consisted of an MRI brain which was obtained at age three due to speech delays and was unremarkable for significant findings. Despite the developmental delays together with physical abnormalities, no genetic testing had been pursued.

Upon our initial evaluation in the clinic, a complete psychiatric review of the systems was performed to clarify her diagnosis. Parents reported behaviors that appeared consistent with ASD, including sensory issues (sensitivity to food texture and avoidance of loud noises), inflexible and rigid behaviors, fixations, stimming behavior (hand flapping, tip-toe walking), and difficulties with socio-emotional reciprocity. The patient also displayed symptoms consistent with ADHD including limited attention span, difficulty organizing tasks, distractibility, fidgety behavior, poor frustration tolerance, and impulsivity. The patient received an official diagnosis of both ADHD and ASD, and she was initiated on methylphenidate hydrochloride (HCl) 20 mg daily and clonidine 0.1 mg nightly to target her emotional lability, impulsivity, anger, and aggression. She was also referred to neuropsychiatric testing and applied behavior analysis (ABA). Additionally, a buccal swab was collected and sent for NextStepDx PLUS Whole Exome Sequencing with an in-office kit from Bionano Laboratories (San Diego, California, US).

Genetic testing results were obtained prior to the follow-up visit about two months later. The results revealed patient was heterozygous for three different sequence variants (KMT2E, ARID1A, and GRIN2D) that were consistent with autosomal dominant genetic disorders (O’Donnell-Luria-Rodan syndrome, Coffin-Siris syndrome, and GRIN2D-related developmental and epileptic encephalopathy). All three of these disorders were associated with developmental delays, ASD, and multiple other medical comorbidities most notable for epilepsy, hypotonia, and GI disturbances. Please refer to Table 1 for full details related to medical comorbidities.

**Table 1:** Genetic Testing Report The results were obtained from from NextStepDx PLUS Whole Exome Sequencing from Bionano Laboratories (San Diego, California, United States), which included sequence variants, associated syndromes, and behavioral/medical comorbidities including GDD (global developmental delay), ID (intellectual disability), and ASD (autism spectrum disorder).

Affected Gene	DNA Change	Protein Change	Zygosity	Associated Syndrome	Associated Symptoms
KMT2E (NM_182931.3)	c.4890_4892dup	p.Pro1634dup	Heterozygous	O’Donnell-Luria-Rodan syndrome (Autosomal dominant)	GDD, ID, ASD, epilepsy, macrocephaly, hypotonia, GI abnormalities
ARID1A (NM_006015.6)	c.5496G>C	p.Arg1833Pro	Heterozygous	Coffin-Siris syndrome (Autosomal dominant)	GDD, ID ASD, speech impairment, hypotonia, feeding difficulties, seizures, vision and hearing abnormalities, coarse facial features
GRIN2D (NM_000836.4)	c.58_60dup	p.Leu20dup	Heterozygous	GRIN2D-related developmental and epileptic encephalopathy (Autosomal dominant)	GDD, ID, hypotonia and spasticity, movement disorders (dystonia, dyskinesia, chorea), ASD, sleep disorders, visual impairment, feeding difficulties, recurrent vomiting and constipation, epilepsy

During the follow up visit, the results of the genetic testing were explained to the patient and family and all questions were answered about the results and prognosis. It was emphasized that patient was at an elevated risk for seizures with these different conditions, and after discussion with family, both provider and parents agreed with preemptive referral to pediatric neurology for evaluation and further management. The patient’s parents discussed that medication so far had reduced frequency of behavioral outbursts, but the patient still experienced some breakthrough periods of agitation as the medication would start to wear off. Additionally, there was concern of oversedation at night. Nighttime clonidine dose was changed to 0.05 mg nightly to help with sedation. Additionally, parents were advised to give clonidine 0.05 daily pro re nata (PRN), or as needed, during severe behavioral outbursts. The patient continues to follow up with the outpatient child and adolescent clinic for medication management.

## Discussion

The genetic testing results from this case highlight the importance of genetic testing early in the diagnostic workup of ASD. This case also illustrates how genetic screening can be conveniently completed in the office during the initial evaluation for ASD. Prior to undergoing testing, the patient’s family was unaware that a genetic mutation could be the source of their daughter’s behavioral and physical symptoms. Table 1 shows that three different genetic mutations likely play a role in the patient’s developmental delays as well as her medical comorbidities including speech delays and visual disturbances. The genetic results also informed the patient and her family about medical complications that may arise in the future and how to prepare for them.

One of the more serious potential complications this patient may experience later in life is epilepsy. Females with O’Donnell-Luria-Rodan syndrome have a 43% chance of developing epilepsy at some point within their lifetime [12]. Individuals with Coffin-Siris syndrome and GRIN2D-related developmental and epileptic encephalopathy are also at an elevated risk for a seizure disorder [13,14]. Given all three of these conditions are associated with seizures, the patient is likely to suffer from seizures at some point in her lifetime. Without receiving the genetic testing, both the patient and her family would have been completely unaware of this potential complication and would likely have been distressed by the sudden development of seizures. With the genetic results, the family has been able to establish care with outpatient pediatric neurology and prepare for the development of this complication.

The discussed case further highlights barriers to genetic testing that can occur within the primary care setting. Although the patient had been diagnosed with ASD three years prior to clinic presentation, genetic testing had never been pursued. It is unclear if genetic testing had been discussed with or offered to the family in the past. Unfortunately, delays or even failure to receive genetic testing in children with developmental disabilities is not uncommon. Surveys have been conducted to determine parent’s understanding and willingness for their children with ASD to receive genetic testing, and results have shown a vast majority (87%) express a desire to pursue genetic testing [15]. Despite this eagerness, the number of patients who receive testing is abysmal. Recent studies estimate that the percentage of patients in the US with an ASD diagnosis that complete genetic resting is roughly 22% [16]. These statistics are alarming as they indicate numerous families are not receiving easily accessible information about their child’s health that could heavily impact the treatment course.

The explanation for this gap is of course multifactorial, and the main contributors that limit genetic testing include poor genetic literacy, financial concerns, and failure of medical providers to educate families about the benefits of testing and make referrals [17]. Genetic literacy refers to the public’s understanding of the role genetics play in a disease process and how determining an underlying genetic cause of a disorder may vastly impact health outcomes. Unfortunately, a knowledge gap does exist when comparing social and economic factors. In general, underserved populations in the US are less likely to appreciate the potential benefits of genetic testing and are more likely to forego these resources [18]. The cost of genetic analysis is also a commonly cited factor for foregoing genetic testing. Although insurance companies are increasing coverage for genetic analysis, this remains highly variable, and families without health insurance are even less likely to pay the out-of-pocket fee. Finally, systematic reviews have identified that providers often serve as a barrier to genetic testing. A 2021 US survey indicated that only 54.9% of child and adolescent psychiatrists had ordered a genetic test for a patient with ASD within the last 12 months [19]. Given that the current consensus among the psychiatric community is that genetic testing should be pursued in all cases of ASD, this statistic is concerning. Therefore, a change in the standard approach to genetic testing needs to be made to ensure quicker and more consistent genetic testing for children with ASD.

A POCT model is a potential avenue to help ensure more patients are offered these resources. POCT involves completing the recommended testing at the point of care, often an outpatient doctor’s office. This serves as a contrast to the previous model of referring patients to an alternative location for testing. This model has become increasingly popular with genetic screening for cancers, and it has been found that implementing POCT has doubled the rates of patients completing such screenings [20]. Furthermore, the POCT model encourages providers to discuss testing benefits with patients directly and offers a more personalized approach based on the patient’s genetic literacy. With these benefits together with the convenience of collecting samples with buccal swab kits in the office, we believe the POCT approach should be made a gold standard during the first encounter for ASD. We recommend that POCT be more widely adopted not only within the psychiatric community but also that this method be made the standard when working up patients for ASD in any primary care setting.

## Conclusions

This case highlights that integrating genetic evaluation into the diagnostic process for ASD and other developmental disorders can immensely help with a better understanding of its etiology, tailoring interventions, and supporting affected families and their children’s future. With advances in access to genetic testing, utilization of a POCT model for the evaluation of developmental disorders should be standardized and will allow for better patient and family engagement in the course of the disorder and its management.
